# PSTP-3,5-Me Inhibits Osteoclast Differentiation and Bone Resorption

**DOI:** 10.3390/molecules24183346

**Published:** 2019-09-14

**Authors:** Eunjin Cho, Zhihao Chen, Jinkyung Lee, Sunwoo Lee, Tae-Hoon Lee

**Affiliations:** 1 Department of Oral Biochemistry, Dental Science Research Institute, School of Dentistry, Chonnam National University, Gwangju 61186, Korea; ag8414@gmail.com (E.C.); wlsrud1945@naver.com (J.L.); 2 Department of Molecular Medicine (BK21plus), Chonnam National University Graduate School, Gwangju 61186, Korea; chinaczhihao@gmail.com; 3 Department of Chemistry, Chonnam National University, Gwangju 61186, Korea; sunwoo@chonnam.ac.kr

**Keywords:** osteoclastogenesis, osteoporosis, PSTP, NFATc1

## Abstract

Osteogenesis is an orchestrated process regulated by osteoclastogenesis and osteoblastogenesis. Excessive osteoclastogenesis causes bone diseases, such as osteoporosis. Although a few drugs are effective in osteoporosis treatment, these drugs lead to side effects, including cellulitis, flatulence, and hypocalcemia. In this study, we reported a 2-(*N*-Phenylmethylsulfonamido)-*N*-(2-(phenylthio)phenyl)propanamide (PSTP) compound, PSTP-3,5-Me, as a potential therapeutic agent for osteoporosis. Mouse bone marrow-derived macrophages (BMMs) were differentiated into osteoclasts by receptor activator of nuclear factor kappa B ligand (RANKL) and macrophage colony-stimulating factor (M-CSF) in the presence of PSTP-3,5-Me. PSTP-3,5-Me inhibited osteoclast differentiation by reduced tartrate-resistant acid phosphatase (TRAP)-positive osteoclasts, and suppressed the expression of osteoclast marker genes, such as cathepsin K (*Ctsk*) and TRAP (*Acp5*). We investigated signaling pathways mediated by RANKL and its receptor, RANK, and found that PSTP-3,5-Me inhibits nucleus translocation of nuclear factor of activated T cell cytoplasmic-1 (NFATc1). Moreover, PSTP-3,5-Me inhibited F-actin ring formation and mineral resorption. Overall, our data suggests that PSTP-3,5-Me attenuates osteoclast differentiation by blocking the activation of NFATc1.

## 1. Introduction

Bone remodeling is maintained by a dynamic balance between osteoblasts and osteoclasts [1]. Osteoblasts are derived from bone marrow-derived mesenchymal stem cells and are involved in bone formation [2]. Hematopoietic lineage-derived osteoclasts are multinucleated cells that participate in bone resorption [3]. Disruption of the balance between osteoclastogenesis and osteoblastogenesis leads to bone diseases, such as osteoporosis and osteopetrosis [4]. Osteoporosis manifests in the form of low bone mass due to excessive bone resorption by osteoclasts, while osteopetrosis is characterized by high bone density due to reduced osteoclast activity [5,6].

Osteoclasts are responsible for osteoporosis due to elimination of mineralized bone [7]. BMMs are differentiated into osteoclasts upon stimulation of RANKL and M-CSF, which are secreted by osteoblasts and osteocytes [7,8]. The interaction between RANKL and RANK on the surface of osteoclasts or their precursors triggers the differentiation-related signaling pathways, including NFATc1, p38 mitogen-activated protein kinase (MAPK), extracellular signal-regulated kinase (ERK), Jun N-terminal kinase (JNK), phosphatidylinositol 3-kinase (PI3K)/Akt, and nuclear factor kappa B (NF-kB) [9]. Mature osteoclasts are fused multi-nucleated cells secreting acidic substances, such as TRAP and CtsK, for the degradation of bone surface [10]. NFATc1 acts as a master transcription factor of RANKL-induced osteoclast differentiation by activating TRAP, CtsK, and DC-STAMP expressions [8]. NFATc1 is dephosphorylated by the serine/threonine phosphatase, calcineurin, and then, translocated into the nucleus [11]. In the nucleus, NFATc1 activates the transcription of the target genes involved in osteoclastogenesis. NFATc1 activation must be regulated tightly for bone homeostasis. Reduced expression of NFATc1 leads to defects in osteoclastogenesis [12], and osteoclast-specific NFATc1-knockout mice develop osteopetrosis [13]. Overexpression of NFATc1 in osteoclast precursors induces osteoclast differentiation without RANKL stimulation [14], and exogenous expression of NFATc1 causes excessive bone resorption [15]. Therefore, proper regulation of NFATc1 is critical for preventing bone diseases.

Sulfonamide carrying *N*-phenylthiophenyl groups have been reported to exhibit biological activities, such as anticancer, anti-inflammatory, and antimicrobial [16,17,18] and triarylsulfonamides have been identified as osteoclast inhibitors, and anti-inflammatory agents [19,20]. Among them, *N*-phenyl substituted methylsulfonamido-*N*-(2-(phenylthio)phenyl)propanamide derivatives have been reported to alter the lifespan of eukaryotic organisms [21]. Several substituents, such as alkyl, alkoxy, and halide, have been conjugated with *N*-phenyl group in 2-(*N*-Phenylmethylsulfonamido)-*N*-(2-(phenylthio)phenyl)propanamide (PSTP) to examine their bioactivity. However, the effects of these PSTP compounds on bone remodeling are still unknown. In this study, we analyzed a novel chemical compound, PSTP-3,5-Me, for attenuation of osteoclast differentiation. The osteoclast differentiation and bone resorption activity of PSTP-3,5-Me-treated BMMs were suppressed. PSTP-3,5,-Me also suppressed *CtsK* and *NFATc1* expression levels. Taken together, these results suggest that PSTP-3,5-Me could be a potential therapeutic agent for osteoporosis.

## 2. Results

### 2.1. PSTP-3,5-Me Inhibits Osteoclast Differentiation

**Figure 1:**
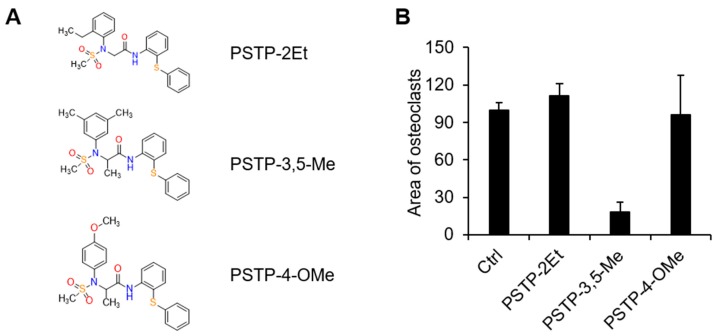
Screening of the PSTP compounds for their effect on osteoclastogenesis. (**A**) The structures of PSTP-2Et, *N*-2-(2-ethylphenyl)-*N*-2-(methylsulfonyl)-*N*-1-[2-(phenylthio) phenyl] glycinamide; PSTP-3,5,-Me, *N*-2-(3,5-dimethylphenyl)-*N*-2-(methylsulfonyl)-*N*-1-[2-(phenylthio)phenyl]alaninamide; PSTP-4-OMe, *N*-2-(4-methoxyphenyl)-*N*-2-(methylsulfonyl)-*N*-1-[2-(phenylthio)phenyl] alaninamide. (**B**) Bone marrow-derived macrophage cells (BMMs) were differentiated into osteoclasts by M-CSF (30 ng/mL) and receptor activator of nuclear factor-kB ligand (RANKL) (50 ng/mL) in the presence of 10 µM PSTP compounds. The area of tartrate-resistant acid phosphatase (TRAP)-positive cells was measured using Image J software.

We performed biological screening with synthetic compounds, containing PSTP structures. There were three commercially available PSTP compounds, PSTP-2Et, PSTP-3,5-Me, and PSTP-4-OMe (Figure 1A). In order to determine the effect of PSTP compounds on osteoclastogenesis, mouse BMMs stimulated with M-CSF (30 mg/mL) and RANKL (50 ng/mL) were incubated with each of the PSTP compounds. After three days, the cells were fixed and stained for TRAP to determine the differentiated multinucleated osteoclasts. Among the three compounds, only PSTP-3,5-Me-treated cells revealed a significant decrease in the abundance of osteoclasts by TRAP staining (Figure 1B). Therefore, we employed PSTP-3,5-Me on osteoclastogenesis.

**Figure 2:**
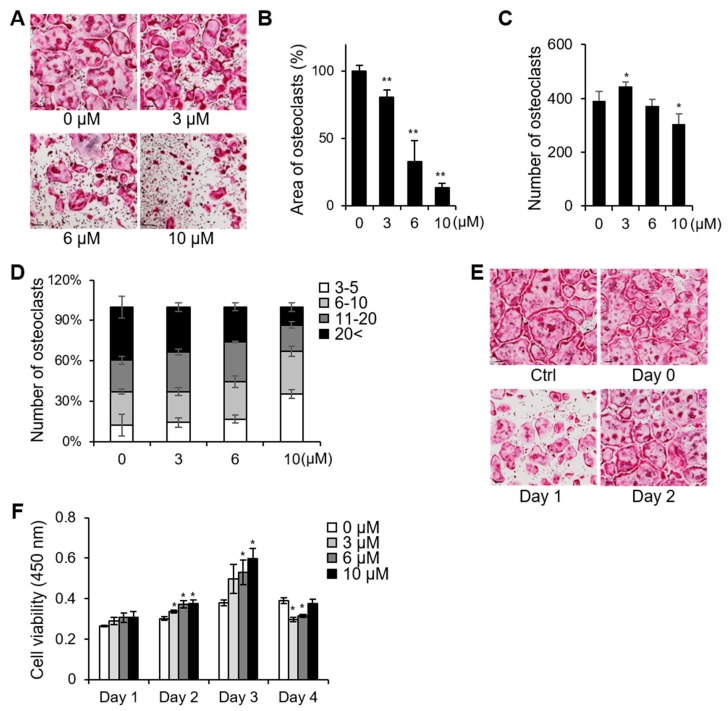
PSTP-3,5-Me inhibited osteoclast differentiation. (**A**) BMMs were differentiated into osteoclasts in the presence of 3, 6, or 10 µM PSTP-3,5-Me for three days. TRAP staining was performed to determine differentiated osteoclasts. Scale bar, 100 µm. The area of TRAP-positive cells was measured using Image J software (**B**), and the number of TRAP-positive cells was counted (**C**), *p < 0.05, **p < 0.01. (**D**) TRAP-positive multinucleated cells harboring more than three nuclei were counted. The percentage of cells with the indicated range of nuclei per cells was calculated. (**E**) PSTP-3,5-Me (6 µM) was added at indicated time/day during osteoclast differentiation. The cells were fixed on day 3 and stained for TRAP activity. Scale bar, 100 µm. (**F**) Cell viability was assessed after treatment with PSTP-3,5-Me during osteoclast differentiation for four days. *p < 0.01.

First, we examined the effects of different concentrations of PSTP-3,5-Me on osteoclastogenesis to determine the half maximal inhibitory concentration (IC_50_) (Figure 2A). Based on the area of TRAP-positive osteoclasts, IC_50_ of PSTP-3,5-Me was approximately 6 µM (Figure 2B). Although the number of differentiated osteoclasts was significantly decreased only after treatment with 10 µM PSTP-3,5-Me, the percentage of giant cells possessing more than 20 nuclei was decreased in the group treated with 6 µM PSTP-3,5-Me (Figure 2C,D). To determine which stage of osteoclastogenesis was inhibited by PSTP-3,5-Me, BMMs were treated with PSTP-3,5-Me for 24 h during osteoclast differentiation on days 0, 1, and 2 and then, the cells were fixed on day 3 (Figure 2E). PSTP-3,4-Me suppressed osteoclastogenesis at the middle stage, whereas treatment with PSTP-3,5-Me at an early or late stage did not affect osteoclastogenesis. These results suggest that PSTP-3,5-Me was much less effective during the initiation of osteoclastogenesis. Interestingly, low concentration of PSTP-3,5-Me (3 µM) enhanced the number of differentiated osteoclast cells (Figure 2C). To investigate whether the reduced differentiation was due to potential cell toxicity, cell viability assay was performed. However, PSTP-3,5-Me treatment revealed increased cell viability compared to controls (Figure 2F). Thus, the results suggest that PSTP-3,5-Me inhibits osteoclast differentiation but not via induction of cell toxicity.

### 2.2. PSTP-3,5-Me Inhibits Osteoclast Differentiation Mediated by Reduced CtsK and NFATc1 Expressions

**Figure 3:**
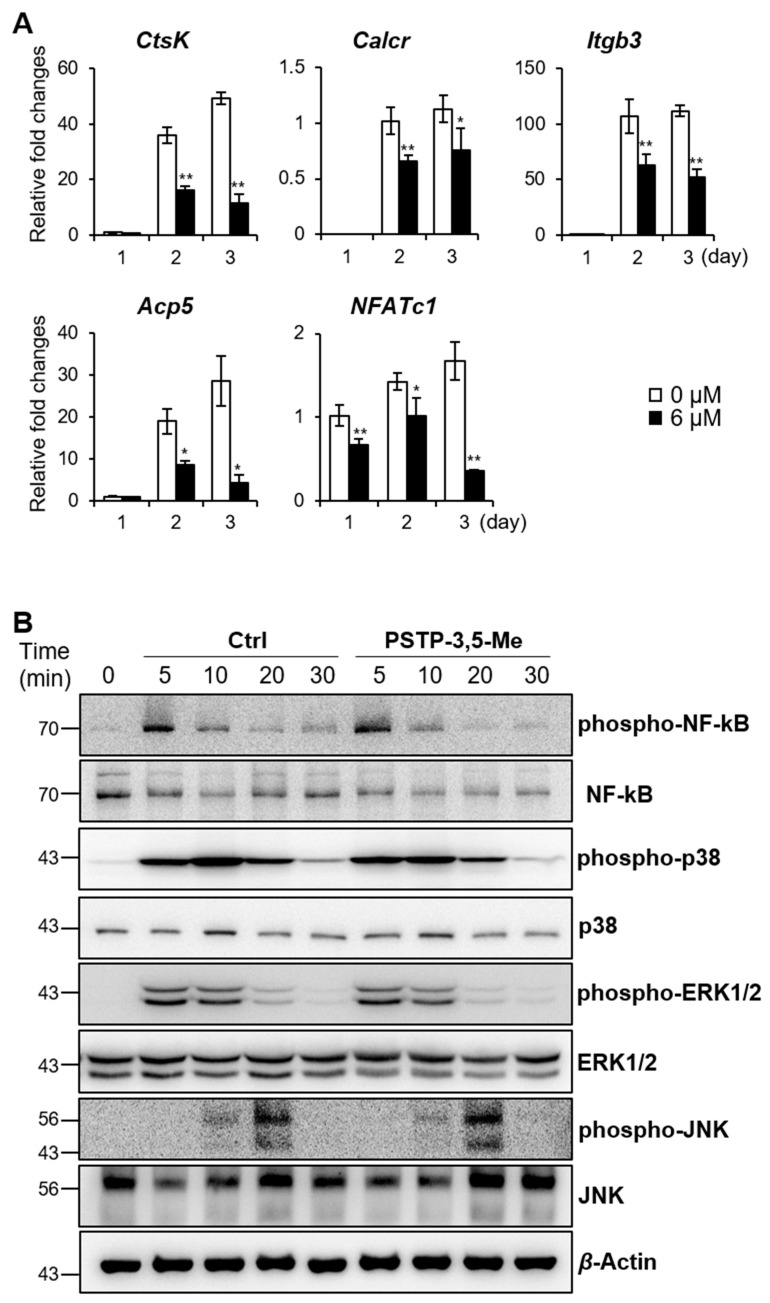
CtsK and NFATc1 expression levels were decreased during osteoclastogenesis by PSTP-3,5-Me treatment. (**A**) mRNA expression levels of osteoclast-specific markers were determined by RT-PCR in 0 or 6 µM PSTP-3,5-Me-treated cells. *p < 0.05, and **p < 0.01 indicate the statistically significant difference between non-PSTP-3,5-Me-treated groups (0 µM) and PSTP-3,5-Me-treated groups (6 µM) on each day. Mean ± standard error. (**B**) Western blotting was performed to determine phosphorylation of NF-kB, p-38, ERK1/2, and JNK. Cells were pre-treated with 6 µM PSTP-3,5-Me or vehicle (Ctrl) for 2 h, and then treated with RANKL for the indicated times. β-Actin expression level was used as a loading control.

We examined the expressions of genes involved in osteoclastogenesis for further validation of the inhibitory effect of PSTP-3,5-Me (Figure 3A). qRT-PCR analysis revealed that *CtsK*, calcitonin receptor (*Calcr*), integrin beta 3 (*Itgb3*), *Acp5*, and *NFATc1* expression levels were increased during osteoclastogenesis in the control group (0 µM). However, the expression levels of these genes were significantly decreased in PSTP-3,5-Me treatment compared to controls. Interestingly, *DC-STAMP* and *OC-STAMP* expression levels, which regulate cell fusion during osteoclastogenesis [22,23], were not altered during differentiation compared to controls (Figure S1).

RANKL-RANK-mediated signaling cascade activates MAPK and NF-kB pathways during osteoclastogenesis [9]. We, therefore, evaluated the phosphorylation of the signaling molecules downstream of RANK, including NF-kB, p38, ERK, and JNK (Figure 3B). BMMs were incubated with PSTP-3,5-Me or vehicle for 2 h, and then, subjected to RANKL stimulation for the indicated time periods. However, there were no significant changes between control and PSTP-3,5-Me-treated samples. These data suggest that PSTP-3,5-Me does not affect early signaling cascade in osteoclastogenesis.

### 2.3. PSTP-3,5-Me Suppresses Nuclear Translocation of NFATc1

**Figure 4:**
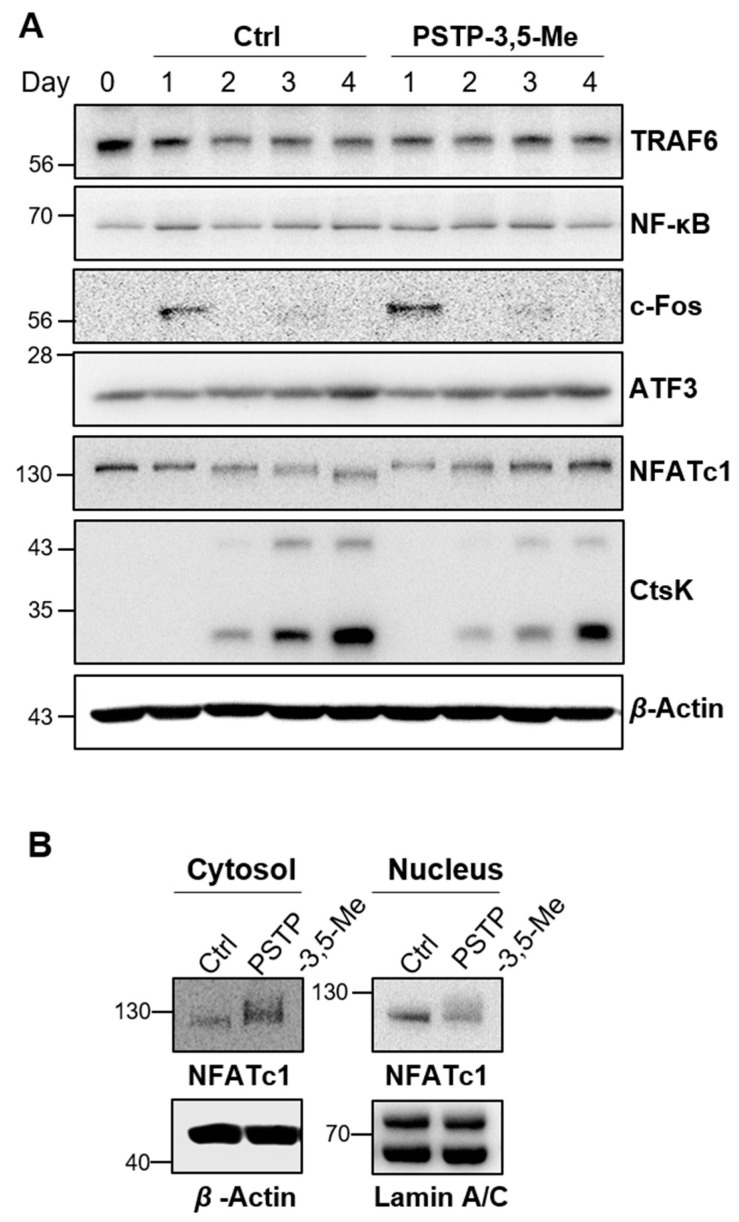
NFATc1 translocation was suppressed by PSTP-3,5-Me. (**A**) RANK-mediated signaling was determined in the presence or absence of 6 µM PSTP-3,5-Me. The protein expression levels of TRAF6, NF-kB, c-Fos, ATF3, NFATc1, and CtsK were analyzed by western blotting, and β-actin was used as a loading control. (**B**) Cytosolic and nuclear proteins were extracted from BMMs with or without PSTP-3,5-Me to determine translocation of NFATc1. β-Actin and Lamin A/C were used as loading controls of cytosolic and nuclear proteins, respectively.

We next examined osteoclast differentiation pathways following RANK activation. The expression levels of TRAF6, NF-κB, c-Fos, and ATF3 were not altered between control and PSTP-3,5-Me treated cells during osteoclast differentiation (Figure 4A and Figure S1). However, NFATc1 expression levels were gradually increased during osteoclast differentiation following PSTP-3,5-Me treatment, while its expression was down-regulated in control cells. Interestingly, NFATc1 protein size was partially smaller at day 4 in control cells. However, this small size band was not detected in PSTP-3,5-Me-treated cells (Figure 4A). NFATc1 is activated by RANKL stimulation via dephosphorylation and nuclear translocation [24]. Therefore, we hypothesized that dephosphorylated NFATc1 was observed in controls. However, its dephosphorylation was blocked by PSTP-3,5-Me. To confirm our hypothesis, we isolated cytosolic and nuclear proteins separately on day 3 of osteoclast differentiation in the control or PSTP-3,5-Me-treated cells (Figure 4B). Cytosolic NFATc1 levels were increased, whereas nucleus NFATc1 levels were decreased in the PSTP-3,5-Me-treated cells compared to controls. These results indicated that PSTP-3,5-Me might inhibit nucleus translocation of NFATc1. In addition, reduced expression of CtsK was observed in PSTP-3,5-Me-treated cells compared to controls. Taken together, a decrease in nuclear localization of NFATc1 leads to lower expression of CtsK, and finally, suppresses complete differentiation of the osteoclast.

### 2.4. PSTP-3,5-Me Inhibits Actin-Ring Formation and Bone-Resorption Activity

**Figure 5:**
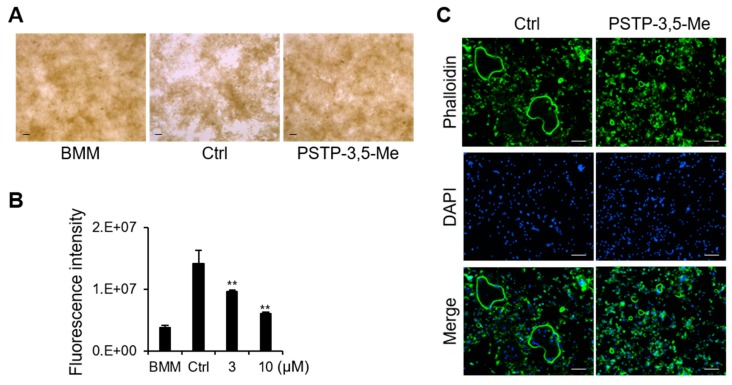
Osteoclast activity was inhibited by PSTP-3,5-Me. (**A**) Bone resorption assay was performed on BMMs with vehicle (Ctrl) or PSTP-3,5-Me treatment. The resorption pit area is indicated as white empty spaces in the picture. (**B**) Fluorescence intensity represented resorption activity in the presence of RANKL and 0, 3, or 10 µM PSTP-3,5-Me; n = 3. BMM is osteoclast precursor cells cultured in only M-CSF. **p-value < 0.01 is considered statistically significant compared to the Ctrl. (**C**) Actin-ring formation was determined by phalloidin antibody in the vehicle (Ctrl)- or PSTP-3,5-Me- treated BMMs by RANKL stimulation.

To determine the effect of PSTP-3,5-Me on osteoclast function via bone resorption activity, BMMs were cultured for five days with or without PSTP-3,5-Me. The resorption areas were detected following M-CSF and RANKL treatment (Ctrl, Figure 5A). However, the resorption areas were not observed following the addition of PSTP-3,5-Me (Figure 5A). We also measured the bone resorption activity using fluoresceinamine-labeled chondroitin sulfate (Figure 5B). As shown in Figure 5A, resorption activity was significantly reduced following PSTP-3,5-Me treatment in a dose-dependent manner. Actin-ring formation is a visual phenotype of mature osteoclasts for osteoclast bone resorption [25]. In the presence of PSTP-3,5-Me, small-sized actin-ring structure was detected, whereas control cells showed distinct actin-ring formation (Figure 5C). These results suggest that PSTP-3,5-Me inhibits mature osteoclast formation, along with a reduction in bone resorption activity.

### 2.5. PSTP-3,5-Me Does Not Affect Osteoblast Differentiation

To determine whether PSTP-3,5-Me is an osteoclast-specific inhibitory compound or is effective in osteoblastogenesis, we examined differentiation of osteoblast cells. Mouse calvarial cells were differentiated into osteoblast cells upon Bmp2 treatment in the presence or absence of PSTP-3,5-Me. Osteoblast differentiation was not affected by treatment with 3 or 6 µM PSTP-3,5-Me, which exhibited an inhibitory effect on osteoclastogenesis (Figure S2). We observed that PSTP-3,5-Me treatment did not alter Alp and Runx2 expression levels compared to controls (data not shown). These data suggest that PSTP-3,5-Me only blocks osteoclast differentiation without any changes to osteoblast differentiation.

## 3. Discussion

In this study, we examined the effects of PSTP derivative compounds on osteoclastogenesis. PSTP compounds exhibit anticancer and anti-inflammatory effects, but their roles in osteoclastogenesis are not well studied. We screened several PSTP compounds and found that only PSTP-3,5-Me exhibited an inhibitory effect on osteoclastogenesis via regulation of *NFATc1* and *CtsK* expressions. We further speculated that PSTP-3,5-Me inhibits nuclear translocation of NFATc1 during osteoclastogenesis.

Osteoporosis is observed in postmenopausal women and older persons worldwide, and it leads to bone fragility and increased susceptibility to fracture [26,27]. Osteoporosis is caused by excessive bone resorption or inadequate bone regeneration due to an imbalance in bone remodeling [28]. Excessive bone degradation by osteoclasts occurs due to estrogen deficiency in women and age-related increases in oxidative stress [5]. Treatments for osteoporosis are focused on anti-resorptive drugs or anabolic drugs that suppress bone resorption and enhance bone formation, respectively [29]. Anti-resorptive drugs include calcitonin, estrogen, and selective estrogen receptor modulators, bisphosphonates, and anti-RANKL antibodies, whereas anabolic drugs include parathyroid hormone and sclerostin inhibitors. However, almost all these drugs have side effects, including osteonecrosis of the jaw, hypocalcemia, and gastrointestinal disorders due to the long-term treatment [30,31,32,33]. Therefore, new therapeutic drugs for treatment of osteoporosis are required.

As shown in Figure 2, low concentration of PSTP-3,5-Me enhanced osteoclast differentiation. However, the number of giant osteoclasts having more than 20 nuclei was slightly reduced in PSTP-3,5-Me-treated cells compared to controls. Although the number of cells was increased, PSTP-3,5-Me blocked full differentiation of the osteoclast. The initiation signaling pathways of osteoclastogenesis are mediated by the phosphorylation of JNK, p38, and NF-kB [8]. However, the phosphorylation levels of these molecules were not altered by PSTP-3,5-Me treatment. RANK activates activator protein-1 (AP-1) transcription factor, which consists of heterodimers of Fos, Jun, and activating transcription factor (ATF) family proteins as an early process to activate its downstream signaling proteins [8,34]. PSTP-3,5-Me did not affect c-Fos and ATF3 expression levels. Therefore, we speculated that PSTP-3,5-Me inhibits osteoclastogenesis in the middle stage during osteoclast fusion process. We examined the effects of PSTP-3,5-Me treatment at different stages on osteoclast differentiation. The osteoclast formation was not altered when PSTP-3,5-Me was treated at an early stage or late stage, while it was slightly reduced at the middle stage treatment. 

The NFAT gene family consists of five members of the Rel/NF-kB family [35]. The NFAT family regulates many biological processes, including immune response, cardiac valve formation, muscle hypertrophy, and osteoclast differentiation [14,35,36,37,38,39]. NFAT1-4 proteins are dephosphorylated by the calcium/calmodulin-dependent phosphatase, calcineurin [35]. Dephosphorylated NFAT protein exposes its nuclear localization signal by conformational changes, and then, moves into the nucleus from the cytosol [8,12,36]. During osteoclastogenesis, NFATc1 accumulates at the *NFATc1* promoter indicating the autoamplification mechanism to elevate the transcription of NFATc1 target genes [34]. At the late stage of osteoclastogenesis, NFATc1 is rephosphorylated by GSK3, resulting in translocation from nucleus to cytoplasm, which leads to the termination of signaling, and then, it is degraded through ubiquitination [24,38,40]. Increased NFATc1 levels in the cytoplasm by PSTP-3,5-Me treatment may be mediated by a reduction in ubiquitination, leading to reduced degradation of NFATc1. We should have analyzed the phosphorylation levels of NFATc1. However, PSTP-3,5-Me treatment revealed a bigger size of NFATc1 compared to controls, suggesting that phosphorylated NFATc1 is maintained by PSTP-3,5-Me. Moreover, nuclear NFATc1 size was slightly smaller than that of the cytosolic NFATc1, indicating that dephosphorylated NFATc1 is localized to the nucleus (Figure 4B). Dephosphorylated NFATc1 expression was reduced following PSTP-3,5-Me treatment. These results indicated other possibilities. For example, PSTP-3,5-Me inhibits dephosphorylation of NFATc1 or PSTP-3,5-Me induces phosphorylation of NFATc1. In future studies, it needs to be confirmed whether PSTP-3,5-Me inhibits calcium uptake or calcineurin activity.

NFATc1 is a master regulator of osteoclastogenesis. Nuclear NFATc1 binds to the DNA with its binding partner, AP-1 [8]. This transcriptional complex regulates transcription of osteoclast specific markers, including TRAP, OSCAR, and CtsK, which are important for the activation of mature osteoclasts. We found that *CtsK, Calcr, Itgb3*, and *Acp5* expression levels were suppressed during osteoclastogenesis by PSTP-3,5-Me treatment. However, *DC-STAMP* and *OC-STAMP* expression levels were not suppressed by PSTP-3,5-Me. NFATc1 regulates osteoclast migration and adhesion via direct regulation of DC-STAMP [41]. However, DC-STAMP expression is regulated by other transcription factors, such as positive regulation factors (c-Fos, PU.1, and NF-kB) and negative regulation factors (Bcl1 and Blimp1) [42,43]. Indeed, NFATc1 nuclear translocation and protein expression are regulated by the levels of DC-STAMP [44]. It is suggested that DC-STAMP expression is controlled not only by NFATc1, but also by other regulators. It is possible that low levels of NFATc1 are sufficient to induce DC-STAMP expression following PSTP-3,5-Me treatment. It must be confirmed in future studies whether PSTP-3,5-Me regulates transcriptional activity of NFATc1.

Our data showed that actin-ring formation and bone-resorption activity were suppressed by PSTP-3,5-Me treatment compared to that in the controls. Some actin-rings were formed in PSTP-3,5-Me- treated BMMs although the size was much smaller than that of those in control cells. The small-sized actin-ring structure contained a lower number of nuclei compared to controls. These results corroborated TRAP staining data in Figure 2D, that PSTP-3,5-Me-treated BMMs develop into osteoclasts containing a smaller number of nuclei than the control BMMs.

Taken together, our results suggest that PSTP-3,5-Me blocks translocation of NFATc1 during differentiation. We showed that the translocation of NFATc1 was critical for the maturation of osteoclasts. In addition, blocking the NFATc1 translocation was sufficient to prevent osteoclast maturation and bone resorption, although alteration in NFATc1 localization is inadequate for regulating expression of all marker genes. These findings suggest that PSTP-3,5-Me could be a potential therapeutic agent against osteoporosis.

## 4. Materials and Methods 

### 4.1. Animal Experiments

All the mice were housed in a specific pathogen-free facility, following the guidelines provided in the Guide for the Care and Use of Laboratory Animals (Chonnam National University, Gwangju, Korea). Adult female C57BL/6J mice (six to eight weeks old) and neonatal mice (three days old) were purchased from DBL Co. (Eumseong, Chungcheongbuk-do, Korea). All the animal experiments were approved by IACUC at Chonnam National University (Approval number CNU IACUC-YB-2017-70).

### 4.2. Osteoclast Differentiation and TRAP Staining

Bone marrow macrophage cells (BMMs) were isolated from femurs and tibias of 8- to 10-week-old C57BL/6J females, as described previously [45]. Briefly, the cells were cultured in alpha minimal essential medium (a-MEM, Thermo Fisher Scientific, Waltham, MA, USA) supplemented with 10% FBS containing 30 ng/mL M-CSF (PeproTech, NJ, USA) for three days. To differentiate into osteoclasts, BMMs were incubated in 30 ng/mL M-CSF and 50 ng/mL RANKL (PeproTech, NJ, USA) containing medium for three to four days. The differentiated cells were fixed on day 3 or day 4 with 4% formaldehyde (Duksan, Korea) with 10 min incubation at room temperature. The fixed cells were stained for tartrate-resistant acid phosphatase (TRAP) activity (Cosmo Bio Co., Ltd., Tokyo, Japan). TRAP-positive osteoclast cells with more than three nuclei were counted, and the area of TRAP-positive cells was measured using Image J software.

### 4.3. Cell Viability Assay

BMMs were cultured in the presence of M-CSF and RANKL in 96-well plates (4 × 10^4^ cells/well). The cells were co-cultured in 0 µM, 3 µM, 6 µM, or 10 µM PSTP-3,5-Me. Cell cytotoxicity assay was performed on days 1, 2, 3, and 4 during differentiation using the EZ-Cytox Cell viability assay kit (Daeil Lab Service Co., Ltd., Cheongwon, Chungcheonbuk-do, Korea).

### 4.4. Quantitative Real-Time PCR (qRT-PCR)

Total RNA was extracted from the BMMs using TRIzol reagent (Thermo Fisher Scientific, MA, USA), as described previously [46]. The complementary DNAs were synthesized using Verso cDNA synthesis kit (Thermo Fisher Scientific, Waltham, MA, USA). Then, quantitative PCR (qPCR) was performed using the QuantStudio 3 real-time PCR system (Applied Biosystems, Waltham, MA, USA) with Power SYBR Green PCR Master Mix (Applied Biosystems, CA, USA). The following primers were used: NFATc1 F-CCCGTCACATTCTGGTCCAT; NFATc1 R-CAAGTAACCGTGTAGCTCCACAA; CtsK F-GGACGCAGCGATGCTAACTAA; CtsK R-CAGAGAGAAGGGAAGTAGAGTTGTCACT; Calcr F-CCTTCCAGAGGAGAAGAAACC; Calcr R-GGAGATTCCGCCTTTTCAC; Itgb3 F-ACAGAGCGTGTCCCGTAATC; Itgb3 R-GTCTTCCATCCAGGGCAATA; Acp5 F-CAGCTGTCCTGGCTCAAAA; Acp5 R-ACATAGCCCACACCGTTCTC; DC-STAMP F-GGGAGTCCTGCACCATATGG; DC-STAMP R-AGGCCAGTGCTGACTAGGATGA; OC-STAMP F-CAGAGTGACCACCTGAACAAACA; OC-STAMP R-TGCCTGAGGTCCCTGTGACT; TRAF6 F-AAAGCGAGAGATTCTTTCCCTG; TRAF6 R-ACTGGGGACAATTCACTAGAGC; RANK F-TCGTCCACAGACAAATGCAAA; RNAK R-GTGTGCTTCTAGCTTTCCAAGG; c-Fos F-CGAAGGGAACGGAATAAGATG; and c-Fos R-GCTGCCAAAATAAACTCCAG. All qPCR experiments were performed in duplicates, and 18S was used as a control. The 2^−ΔΔCt^ method was used for data analysis.

### 4.5. Western Blotting

Cells were lysed in RIPA lysis buffer (Thermo Fisher Scientific, Waltham, MA, USA) containing protease and phosphatase inhibitor cocktails (Sigma-Aldrich, St. Louis, MO, USA). Nuclear proteins were extracted using NE-PER nuclear and cytoplasmic extraction reagents (Thermo Fisher Scientific), according to the manufacturer’s instructions. The concentration of protein lysates was measured using a BCA protein assay kit (Thermo Fisher Scientific). The proteins were subjected to a SDS-PAGE gel electrophoresis and transferred to PVDF membranes. Then, the membranes were incubated with anti-phospho-Akt (4060), anti-Akt (9272), anti-phospho-NF-kB (3033), anti-NF-kB (4764), anti-phospho-p38 (4511), anti-phospho-ERK1/2 (4370), anti-ERK1/2 (9102), anti-phospho-JNK (9255), anti-JNK (9252), Anti-c-Fos (4384), anti-NFATc1 (8032), or anti-Lamin A/C (2032) antibodies which were purchased from Cell Signaling Technology (Danvers, MA, USA), or anti-Cathepsin K (Santa Cruz Biotech., sc-48353, Dallas, TX, USA), anti-ATF3 (Abcam, ab180842, Burlingame, CA, USA), anti-TRAF6 (Abcam, ab40675), anti-p38 (Novus Biologicals, NB110-96907, St Charles, MO, USA), or anti-β-actin (Sigma-Aldrich, A5441) followed by incubation with horseradish peroxidase (HRP)-conjugated anti-rabbit or anti-mouse secondary antibody purchased from Cell Signaling Technology. All the antibodies were used in a 1:1000 dilution in 5% skim milk as recommended by the manufacturer.

### 4.6. Osteoblast Differentiation and ALP Staining

Primary mouse osteoblasts were isolated from the calvaria of three-day-old C57BL/6J mice [45]. The cells were cultured in α-MEM with 10% FBS and differentiated into osteoblast by addition of 100 ng/mL BMP-2 (Sino Biological, Beijing, China). Different concentrations of PSTP-3,5-Me were treated for 7 or 14 days. Alkaline phosphatase (ALP) staining was performed at day 7 or 14 using NBT-BCIP solution (Sigma-Aldrich).

### 4.7. Bone Resorption Assay

Bone resorption activity was determined using bone resorption assay kit (CosMo Bio, Tokyo, Japan), as described previously [45]. Briefly, BMMs were cultured on a calcium phosphate-coated 48-well plate (2.5 × 10^4^ cells/well) in the presence of M-CSF (30 ng/mL) and RANKL (100 ng/mL) with different concentrations of PSTP-3,5-Me (0, 3, and 10 µM) for six days. The fluorescence intensity of the culture supernatant was analyzed using a fluorescence plate reader, SpectraMax i3x (Molecular Devices, San Jose, CA, USA) with 485 nm and 535 nm wavelengths. The pit area was calculated using Image J software.

### 4.8. Actin Ring Formation

Differentiated osteoclast cells either treated or untreated with 6 µM PSTP-3,5-Me were stained using Alex Fluor 499 Phalloidin antibody (Thermo Fisher Scientific) to label F-actin. DAPI staining was used for counterstaining the nucleus (Thermo Fisher Scientific), and then, visualized using a fluorescence microscope, Lionheart FX (BioTek, Winooski, VT, USA). 

### 4.9. Statistical Analysis

All values were expressed as mean ± standard deviation (SD) of three independent experiments, or as indicated. The difference between vehicle-treated control (Ctrl) and PSTP-3,5-Me-treated groups was analyzed using unpaired two-tailed Student’s *t*-tests.

## Supplementary Materials

The following are available online. Figure S1: mRNA expression of the genes involved in osteoclastogenesis; Figure S2: Effects of PSTP-3,5-Me on osteoblast differentiation.
