# Supplementary material for: PSTP-3,5-Me Inhibits Osteoclast Differentiation and Bone Resorption
Source: Molecules. 2019 Sep 14;24(18):3346. doi: 10.3390/molecules24183346 (PMC6767254; doi:10.3390/molecules24183346)
Supplement: Supplementary file 1 [file molecules-24-03346-s001.pdf]

Supplementary Materials

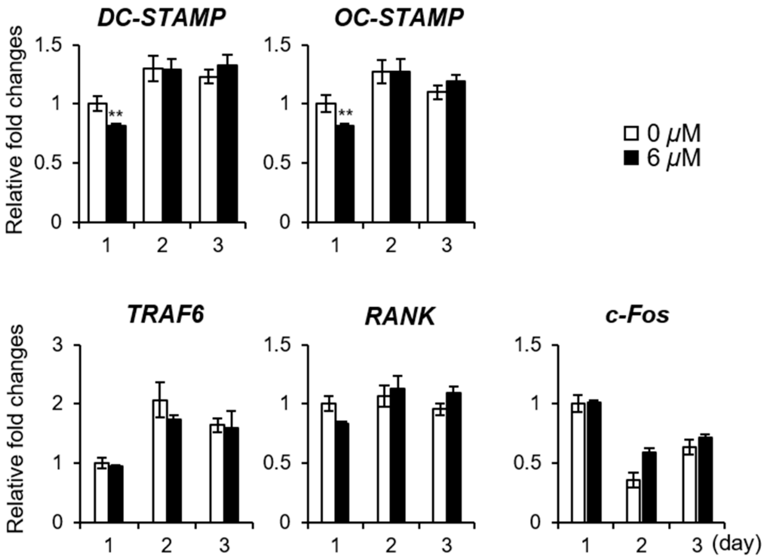

Figure S1. mRNA expression of the genes involved in osteoclastogenesis. The mRNA expression levels were determined by RT-PCR in 0 or 6 μM PSTP-3,5-Me-treated cells. ** $p < 0.01$ indicates the significant difference between non-PSTP-3,5-Me-treated groups (0 μM) and PSTP-3,5-Me-treated group (6 μM) on each day. Values represent the mean ± standard error.

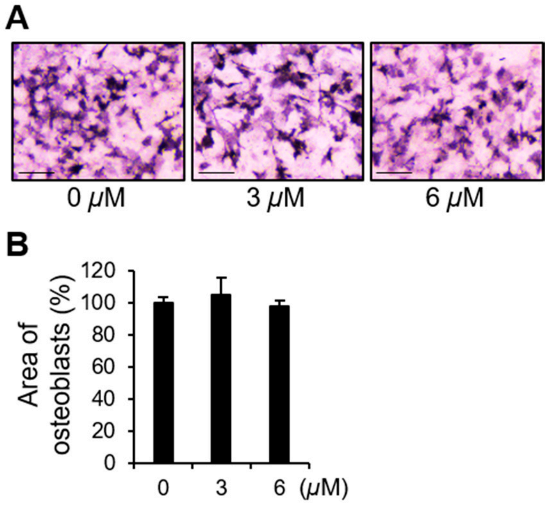

Figure S2. Effects of PSTP-3,5-Me on osteoblast differentiation. Calvarial cells were differentiated into osteoblasts upon stimulation with Bmp2 (100 ng/mL) with or without PSTP-3,5-Me (3 μM or 6 μM) treatment. The cells were cultured for seven days and ALP staining was performed to determine differentiated osteoblasts (**A**). The area of ALP-positive cells was quantified using Image J software (**B**). Scale bar, 100 μM.
